# Constructing a gnotobiotic mouse model with a synthetic human gut microbiome to study host–microbe cross talk

**DOI:** 10.1016/j.xpro.2021.100607

**Published:** 2021-06-12

**Authors:** Alex Steimle, Alessandro De Sciscio, Mareike Neumann, Erica T. Grant, Gabriel V. Pereira, Hiroshi Ohno, Eric C. Martens, Mahesh S. Desai

**Affiliations:** 1 Department of Infection and Immunity, Luxembourg Institute of Health, Esch-sur-Alzette 4354, Luxembourg; 2 Faculty of Science, Technology and Medicine, University of Luxembourg, Esch-sur-Alzette 4365, Luxembourg; 3 University of Michigan Medical School, Ann Arbor, MI 48109, USA; 4 RIKEN Center for Integrative Medical Sciences, Yokohama, Kanagawa 230-0045, Japan; 5 Immunobiology Laboratory, Graduate School of Medical Life Science, Yokohama City University, Yokohama, Kanagawa 230-0045, Japan; 6 Laboratory for Immune Regulation, Graduate School of Medicine, Chiba University, Chiba, Chiba 260-8670, Japan; 7 Odense Research Center for Anaphylaxis, Department of Dermatology and Allergy Center, Odense University Hospital, University of Southern Denmark, 5000 Odense, Denmark

**Keywords:** Immunology, Microbiology, Model Organisms

## Abstract

Reproducible *in vivo* models are necessary to address functional aspects of the gut microbiome in various diseases. Here, we present a gnotobiotic mouse model that allows for the investigation of specific microbial functions within the microbiome. We describe how to culture 14 different well-characterized human gut species and how to verify their proper colonization in germ-free mice. This protocol can be modified to add or remove certain species of interest to investigate microbial mechanistic details in various disease models.

For complete details on the use and execution of this protocol, please refer to Desai et al. (2016).

## Highlights

• Colonization of germ-free mice with a synthetic human gut microbiome
• One-medium-fits-all culturing approach for phylogenetically distinct bacteria
• Rapid and easy-to-perform verification of colonization success
• Useful to study functional aspects of the microbiome in various diseases

## Graphical abstract

**Figure:**
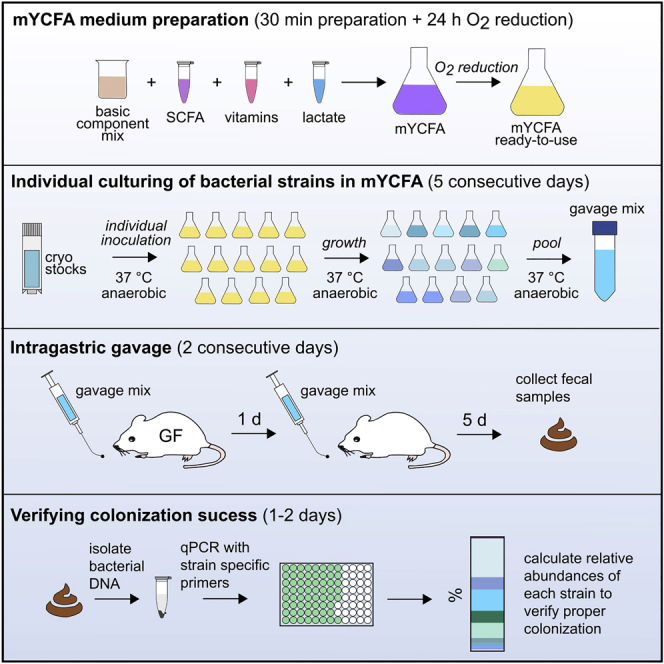

## Before you begin

This protocol describes four different steps: (1) preparation of a custom-made bacterial culture medium kit; (2) cultivation of intestinal commensals in a single medium under anaerobic conditions; (3) preparation of the bacterial mix for intragastric gavage into germ-free (GF) mice; and (4) evaluation of colonization success in these mice.

**Figure 1:**
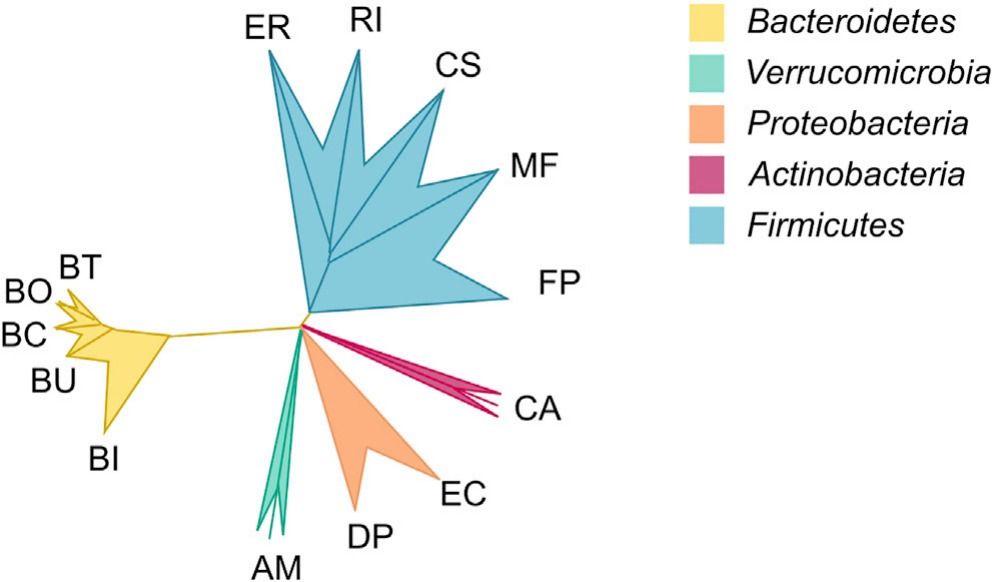
Phylogenetic relation of the 14 human commensal strains featured in this protocol, which are further specified in Table 1.

The following protocol involves the use of 14 different, fully sequenced, human gut commensal species, which were previously used to address the role of microbiome-mediated mucus degradation during enteropathogenic infection in a gnotobiotic mouse model (Desai et al., 2016). We have further optimized the growth conditions of the 14 strains in this protocol. Table 1 summarizes the microbial species used in this protocol and Figure 1 illustrates their broad-level phylogenetic relation. Importantly, all of these strains are available from commercial bacterial collections. However, other strains not mentioned in this list can potentially be used instead of the herein listed strains, or in addition to them, depending on specific research interests and after verification of their ability to grow in the recommended bacterial culture medium. The modified yeast- and short-chain fatty acid-containing culture medium (mYCFA) is based on a protocol previously published by Browne et al. (Browne et al., 2016) and its composition was adapted to the needs of the bacterial strains used in this protocol. Thus, as compared to the original protocol, mYCFA does not contain maltose and cellulose. Additionally, mYCFA is supplemented with *N*-acetyl-D-glucosamine to support growth of the mucin-specialist *Akkermansia muciniphila*. Furthermore, the concentration of sulfate ions is increased 46-fold and sodium lactate is added to support proper culturability of *Desulfovibrio piger*. These changes do not negatively affect growth of the other 12 constituent strains within the 14-member community.

**Table 1:** List of human commensal species

Abbreviation	Strain	Supplier	Cat#	Phylum
AM	*Akkermansia muciniphila*: DMS 22959, type strain	DSMZ	DSM 22959	*Verrucomicrobia*
BC	*Bacteroides caccae*: DSM 19024, type strain	DSMZ	DSM 19024	*Bacteroidetes*
BO	*Bacteroides ovatus*: DSM 1896, type strain	DSMZ	DSM 1896	*Bacteroidetes*
BT	*Bacteroides thetaiotaomicron*: DSM 2079, type strain	DSMZ	DSM 2079	*Bacteroidetes*
BU	*Bacteroides uniformis*: ATCC 8492, type strain	ATCC	ATCC 8492	*Bacteroidetes*
BI	*Barnesiella intestinihominis*: YIT 11860	DSMZ	DSM 21032	*Bacteroidetes*
CS	*Clostridium symbiosum*: DSM 934, type strain, 2	DSMZ	DSM 934	*Firmicutes*
CA	*Collinsella aerofaciens*: DSM 3979, type strain	DSMZ	DSM 3979	*Actinobacteria*
DP	*Desulfovibrio piger*: ATC 29098, type strain	ATCC	ATC 29098	*Proteobacteria*
EC	*Escherichia coli* HS	ATCC	N/A	*Proteobacteria*
ER	*Eubacterium rectale*: DSM 17629, A1-86	DSMZ	DSM 17629	*Firmicutes*
FP	*Faecalibacterium prausnitzii*: DSM 17677, A2-165	DSMZ	DSM 17677	*Firmicutes*
MF	*Marvinbryantia formatexigens*: DSM 14469, type strain, I-52	DSMZ	DSM 14469	*Firmicutes*
RI	*Roseburia intestinalis*: DSM 14610 type strain, L1-82	DSMZ	DSM 14610	*Firmicutes*

To generate the mYCFA medium, we recommend setting up an in-house kit, which consists of five different components. Each component can be prepared and aliquoted in advance to reduce workload on the day of experiment. Each aliquot is designed to yield 100 mL of a ready-to-use mYCFA liquid medium.

Culturing of all strains must be performed at 37°C in an anaerobic chamber, with an atmosphere of 85% N_2_, 10% CO_2_ and 5% H_2_, preferably supported by a palladium catalyst to aid in maintaining the strictly anaerobic conditions. Hypoxic conditions are not sufficient to promote proper growth of all strains; thus, the culture medium and all plastic materials needed for cultivation must be placed in the anaerobic chamber at least 24 h before start of the culturing procedure for proper oxygen reduction in liquids and plastic material.

**Figure 2:**
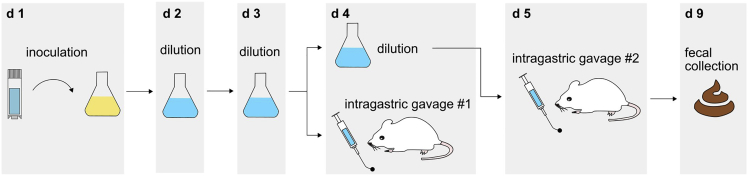
Timeline for colonization of germ-free mice with 14 different human commensals Start with inoculation of each strain separately in mYCFA medium (day 1). Bacterial cultures are diluted one (d 2) and two days (d 3) later. On day 4 (d 4), bacterial suspensions will be mixed for intragastric gavage (lower panel). Remaining bacterial suspensions will be further diluted and incubated for subsequent gavage on day 5 (d 5). Fecal samples can be collected 5 d after initial intragastric gavage (d 9) for DNA isolation and colonization verification.

We also describe, in detail, how to grow these 14 different bacterial strains using the mYCFA medium over a period of 4 days prior to the final gavage (see also Figure 2 for an overview of the experimental timeline). Cultivation of all 14 strains should start 3 d prior to the first gavage. Gavaging should be performed twice on two consecutive days and colonization success can be checked by isolating bacterial DNA from fecal samples that have been collected 5 d after the first gavage. Furthermore, we provide detailed information on how to elucidate success of colonization and offer a variety of tips and solutions for troubleshooting.

Before the culturing procedure begins, we recommend aliquoting all components needed for quick preparation of the ready-to-use mYCFA medium. We describe the preparation of 30 aliquots of each component. This is sufficient for 3 L of ready-to-use medium, which allows for 7 to 8 gavage cycles.

### Preparation of mYCFA basic components (mYCFA kit component 1)

**Timing: 2 h**

The following recipe describes the preparation of 30 aliquots of “mYCFA basic components” powder (for 3 L of ready-to-use liquid medium). Please note that preparation under sterile conditions (i.e., under a laminar flow) is not necessary for this step.

1. In a 1 L plastic or glass beaker, combine the components listed in Table 2 to generate a powder mix of 69.7 g.

**Table 2:** Components of mYCFA basic component powder (kit component 1)

Component	For 100 mL liquid medium (1 aliquot)	For 3 L liquid medium (30 aliquots)	Final concentration
Casitone/Peptone	1 g	30 g	10 g L^−1^
Yeast extract	250 mg	7.5 g	2.5 g L^−1^
NaHCO_3_	400 mg	12 g	4 g L^−1^
Cysteine	100 mg	3 g	1 g L^−1^
K_2_HPO_4_	45 mg	1.35 g	0.45 g L^−1^
NaCl	90 mg	2.7 g	0.9 g L^−1^
MgSO_4_ ∗ 7 H_2_O	419 mg	12.57 g	4.19 g L^−1^
CaCl_2_	9 mg	270 mg	90 mg L^−1^
Resazurin	100 μg	3 mg	1 mg L^−1^
Hemin	1 mg	30 mg	10 mg L^−1^
TOTAL	2.31 g powder	69.7 g powder

***Note:*** This powder mix will be aliquoted into 2.31 g aliquots of powder, which can be stored at 4°C. When solubilized in ddH_2_O (see below), 1 powder aliquot generates 100 mL of liquid medium for immediate use (see below).

2. Mix the powder well by using an approx. 20 cm metal or plastic stick or a spatula.
3. To ensure an equal distribution of all of the solid components, use a mortar to homogenize the powder mix in 10 g batches, creating a smooth, fine-grained powder, and transfer the powder into a fresh 1 L glass or plastic beaker. Continue until the whole powder mix has a fine-grained texture.
4. Again, mix well using an approx. 20 cm metal or plastic stick or a spatula.
5. Aliquot 2.31 g of the fine-grained powder mix into 50 mL screw cap tubes (e.g., a falcon tube).
6. Store the mYCFA basic components powder mix at 4°C for up to 6 months.

***Note:*** we can assure the kit’s stability for up to 6 months; however, we recommend further validation tests for the usage of any components past this period.

### Preparation of mYCFA short-chain fatty acid (SCFA) mix (mYCFA kit component 2)

**Timing: 1 h**

The following recipe describes the preparation of 30 aliquots of “mYCFA SCFA mix” (sufficient for 3 L of ready-to-use medium):

***Note:*** Prepare stocks under a fume hood.

7. Add the components listed in Table 3 to a proper vessel to yield 8.607 mL of the SCFA mix.

**Table 3:** Components of the SCFA mix

SCFA	M_W_	Density	Molarity	Volume to use for 100 mL liquid medium (1 aliquot)	Volume to use for 3 L liquid medium (30 aliquots)	Final concentration in liquid medium
Acetic acid	60 g mol^−1^	1.05 g mL^−1^	17.50 M	189 μL	5657 μL	33 mM
Propionic acid	74 g mol^−1^	0.99 g mL^−1^	13.38 M	67 μL	2018 μL	9 mM
Isobutyric acid	88 g mol^−1^	0.97 g mL^−1^	11.02 M	9 μL	272 μL	1 mM
Isovaleric acid	102 g mol^−1^	0.925 g mL^−1^	9.07 M	11 μL	331 μL	1 mM
Valeric acid	102 g mol^−1^	0.93 g mL^−1^	9.12 M	11 μL	329 μL	1 mM
TOTAL				0.287 mL	8.607 mL
8. Vortex.
9. Filter-sterilize the solution through a 0.22 μm filter.
10. Aliquot 287 μL of this mix into sterile 1.5 mL screw cap tubes.
11. Due to the volatile nature of SCFAs, seal the tubes with parafilm.
12. Aliquots can be stored at 4°C for up to 6 months.

### Preparation of mYCFA vitamin mix (mYCFA kit component 3)

**Timing: 2 h**

The following recipe describes the preparation of 100 mL of the mYCFA vitamin mix. This volume is sufficient for 1000 aliquots of 100 μL. 1 aliquot of 100 μL vitamin mix is used for 100 mL ready-to-use mYCFA medium. Thus, 100 mL of the mYCFA vitamin mix is sufficient for a total of 100 L of ready-to-use mYCFA medium. This large volume ensures it is possible to accurately weigh the small amounts of vitamins needed. We recommend aliquoting 50–100 aliquots of 100 μL under a laminar flow in sterile, light-protected 1.5 mL tubes and storing the remaining solution at 4°C under light-protected conditions.

13. Add the components listed in Table 4 to a 200 mL glass bottle.

**Table 4:** Components of the vitamin mix

Component	For 100 mL mYCFA medium (1 aliquot)	For 100 mL of vitamin stock (1000 aliquots)	Final concentration in mYCFA medium
Biotin	1 μg	1 mg	10 μg L^−1^
Vitamin B12	1 μg	1 mg	10 μg L^−1^
4-Aminobenzoic acid	3 μg	3 mg	30 μg L^−1^
Folic acid	5 μg	5 mg	50 μg L^−1^
Pyridoxine hydrochloride	15 μg	15 mg	150 μg L^−1^
Thiamine hydrochloride	5 μg	5 mg	50 μg L^−1^
(–)-Riboflavin	5 μg	5 mg	50 μg L^−1^
14. Resuspend in 100 mL ddH_2_O and vortex.
15. Filter-sterilize the solution using a 0.22 μm filter.
16. Aliquot 100 μL of this mix into sterile, light-protected 1.5 mL tubes.
17. Aliquots can be stored at 4°C for up to 6 months.
18. Cover the bottle containing the leftover suspension with aluminum foil to block out light and store at 4°C.

### Preparation of D-glucose solution (mYCFA kit component 4)

**Timing: 30 min**

Perform the preparation under a laminar flow.

19. Dissolve 3 g of glucose in 15 mL ddH_2_O to achieve a concentration of 200 mg mL^−1^.
20. Mix well.
21. Filter-sterilize the solution using a 0.22 μm filter.
22. Aliquot 500 μL of this solution into a sterile 1.5 mL tube.
23. Aliquots can be stored at −20°C for up to 6 months.

### Preparation of N-acetyl-D-glucosamine solution (mYCFA kit component 5)

**Timing: 30 min**

Perform the preparation under a laminar flow.

24. Dissolve 3 g of N-acetyl-D-glucosamine in 15 mL ddH_2_O to achieve a concentration of 200 mg mL^−1^.
25. Mix well.
26. Filter-sterilize the solution using a 0.22 μm filter.
27. Aliquot 500 μL of this solution into a sterile 1.5 mL tube.
28. Aliquots can be stored at −20°C for up to 6 months.

### Preparation of 100 mL buffer A (DNA extraction component 1)

**Timing: 20 min**

29. For 100 mL of buffer A, add 1.169 g NaCl, 2.423 g Trizma Base and 4 mL EDTA 0.5 M, pH 8.0 into 200 mL glass bottle and fill *up to* 100 mL with ddH_2_O (see Table 5).

**Table 5:** Components of buffer A

Reagent	For 100 mL of buffer A	Final concentration
NaCl	1.169 g	0.2 M
Trizma Base	2.423 g	0.2 M
EDTA 0.5 M, pH 8.0	4 mL	0.02 M
ddH_2_O	Up to 100 mL	-

30. Filter-sterilize buffer A using a 0.22 μm filter.
31. Buffer A can be stored at RT (room temperature; 20°C–25°C) for up to 6 months.

### Preparation of 3 M sodium acetate (DNA extraction component 2)

**Timing: 15 min**

32. Resuspend 4.08 g sodium acetate in 8 mL ddH_2_O.
33. Adjust pH to 5.2.
34. Fill up to 10 mL with ddH_2_O.
35. Filter-sterilize using a 0.22 μm filter.
36. Solution can be stored at RT for up to 6 months.

### Preparation of 20 mL 20% SDS solution (DNA extraction component 3)

**Timing: 15 min**

37. Resuspend 4.0 g sodium dodecyl sulfate (SDS) in 15 mL ddH_2_O.
38. Filter-sterilize the solution using a 0.22 μm filter.
39. Solution can be stored at RT for up to 6 months.

## Key resources table

**Table:** 

REAGENT or RESOURCE	SOURCE	IDENTIFIER
**Bacterial and virus strains**		

*Akkermansia muciniphila*	DSMZ	DSM 22959
*Bacteroides caccae*	DSMZ	DSM 19024
*Bacteroides ovatus*	DSMZ	DSM 1896
*Bacteroides thetaiotaomicron*	DSMZ	DSM 2079
*Bacteroides uniformis*	ATCC	ATCC 8492
*Barnesiella intestinihominis* YIT 11860	DSMZ	DSM 21032
*Clostridium symbiosum* 2	DSMZ	DSM 934
*Collinsella aerofaciens*	DSMZ	DSM 3979
*Desulfovibrio piger*	ATCC	ATC 29098
*Escherichia coli* HS	ATCC	NR-9280
*Eubacterium rectale* A1-86	DSMZ	DSM 17629
*Faecalibacterium prausnitzii* A2-165	DSMZ	DSM 17677
*Marvinbryantia formatexigens* I-52	DSMZ	DSM 14469
*Roseburia intestinalis* L1-82	DSMZ	DSM 14610

**Biological samples**		

Stools from male and female C57BL/6 mice, age 6–8 weeks, housed under germ-free conditions	N/A	N/A

**Chemicals, peptides, and recombinant proteins**		

Water, Bioscience-Grade, Nuclease-free (500 mL)	Carl Roth	Cat#T143.2
Ethanol for molecular biology (250 mL)	VWR	Cat#1.08543.0250
Proteinase K (10 mL)	QIAGEN	Cat#19133
Buffer ATL (200 mL)	QIAGEN	Cat#19076
Chloroform, 99.8+%, Certified AR for Analysis, Stabilised with Amylene, Fisher Chemical™	Fisher Scientific	Cat#10122190
RNase A (Reagents for GeneJET™ Plasmid Miniprep Kit, Thermo Scientific R1253)	Fisher Scientific	Cat#10202510
Phenol:Chloroform:Isoamyl Alcohol 25:24:1, Saturated with 10 mM Tris, pH 8.0, 1 mM EDTA	Sigma	Cat#P3803
Glass Beads, acid-washed	Sigma-Aldrich	Cat#G1277-500g
Invitrogen™ dNTP Set (100 mM) Solution	Fisher Scientific	Cat#10083252
Platinum™ *Taq* DNA Polymerase	Life Technologies	Cat#10966034
SYBR™ Green I Nucleic Acid Gel Stain, 10,000× concentrate in DMSO	Invitrogen	Cat#S7585
D-(+)-Glucose	Sigma-Aldrich	Cat#G7528
N-Acetyl-D-glucosamine	Sigma-Aldrich	Cat#A3286
Sodium DL-lactate solution, 60% (w/w)	Sigma-Aldrich	Cat#L1375
Resazurin sodium salt	Sigma-Aldrich	Cat#199303
Hemin	Sigma-Aldrich	Cat#51280
Biotin	Sigma-Aldrich	Cat#B4501
Vitamin B12 (cobalamin)	Sigma-Aldrich	Cat#V2876
4-Aminobenzoic acid	Sigma-Aldrich	Cat#A9878
Folic acid	Sigma-Aldrich	Cat#F8758
Pyridoxine hydrochloride	Carl Roth	Cat#T914.1
Thiamine hydrochloride	Sigma-Aldrich	Cat#T4625
(−)-Riboflavin	Sigma-Aldrich	Cat#R9504
Isobutyric acid	Sigma-Aldrich	Cat#58360
Isovaleric acid	Sigma-Aldrich	Cat#129542
Valeric acid	Sigma-Aldrich	Cat#240370
Calcium chloride	Carl Roth	Cat#A119.1
Peptone ex casein	Carl Roth	Cat#8986.2
Yeast extract, micro-granulated	Carl Roth	Cat#2904.2
Sodium hydrogen carbonate	Carl Roth	Cat#HN01.1
L-Cysteine	Carl Roth	Cat#1693.2
di-Potassium hydrogen phosphate	Carl Roth	Cat#6875.1
Sodium chloride	Carl Roth	Cat#3957.1
Acetic acid	Carl Roth	Cat#6755.1
Propionic acid	Carl Roth	Cat#6026.2
Magnesium sulphate heptahydrate	Carl Roth	Cat#P027.1
Gas Mix 5% H_2_, 5% CO_2_, 90% N_2_	Air Liquide	Cat#23160133

**Critical commercial assays**		

DNeasy Blood & Tissue Kit (250)	QIAGEN	Cat#69506

**Experimental models:Organisms/strains**		

C57BL/6 germ-free mice	Taconic Biosciences	Cat#GF-B6

**Oligonucleotides**		

Primers for 14 bacteria strains, see Table 9	Eurogentec	N/A

**Software and algorithms**		

Bio-Rad CFX Manager 3.0	CFX Manager Software	http://www.bio-rad.com

**Other**		

Stericup-GP, 0.22 μm, polyethersulfone, 250 mL, radio-sterilized	Millipore	Cat#SCGPU02RE

## Step-by-step method details

### Part A: Preparation of complete mYCFA bacterial culture medium

**Timing: Preparation 30 min. Oxygen-reduction 24 h.**

Prepared mYCFA medium should not be stored for more than 72 h due to volatility of some of its components, especially SCFAs. When following the culturing instructions described in this protocol, a total of 400 mL of mYCFA is needed to culture 14 strains during a period of 5 days. Thus, we recommend to prepare 200 mL one day prior to initial inoculation (initial inoculation on “d 1” in Figure 2) and another 200 mL one day before initial gavage (initial gavage on “d 4” in Figure 2).

In this step, mYCFA kit components 1 to 5 (see “Before You Begin”) will be used to generate the complete mYCFA medium. In the following instructions, we describe the generation of 100 mL of medium.

1. Dissolve 1 aliquot of the mYCFA basic component mix (kit component 1) in 50 mL ddH_2_O.

**Figure 3:**
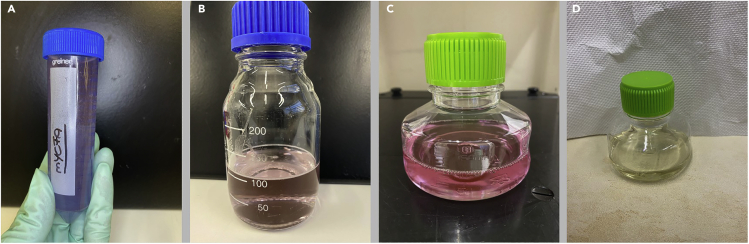
Visual appearance of mYCFA medium during different steps of preparation (A) Blue color of mYCFA basic component mix (kit component 1) dissolved in 50 mL ddH20. (B) Purple color of mYCFA after addition of SCFA. (C) Pink color after pH adjustment to pH 6.8. (D) Oxygen-reduced yellow mYCFA medium, 24 h after transfer into an anaerobic chamber.

***Note:*** Color turns blue immediately (Figure 3A).

2. Transfer the 50 mL into a 200 mL glass bottle.
3. Rinse the 50 mL Falcon tube of the mYCFA basic component mix with an additional 50 mL ddH_2_O and transfer this volume to the glass bottle to achieve a 100 mL solution.
4. Add 1 aliquot of mYCFA SCFA mix (kit component 2).

***Note:*** Color turns purple and bubbles arise (Figure 3B).

5. Add 1 aliquot of mYCFA vitamin mix (kit component 3).
6. Add 1 aliquot of mYCFA D-glucose solution (kit component 4).
7. Add 1 aliquot of mYCFA N-acetyl-D-glucosamine solution (kit component 5).
8. Add 444 μL of 60% sodium lactate solution.
9. Adjust pH value to 6.8. Color turns pink (Figure 3C)
10. Filter-sterilize the complete mYCFA medium using a 0.22 μm filter.
11. Transfer the mYCFA medium into an anaerobic chamber for at least 24 h for oxygen reduction and use the medium within 72 h. Inside the anaerobic chamber, leave the screw cap slightly loosened to aid in oxygen reduction. Once the oxygen is reduced (color change to yellow), the cap can be tightened.

***Note:*** Color of the medium turns from pink to yellow when solution is properly oxygen-reduced and ready to use (Figure 3D).

***Note:*** The medium contains a compound that is very sensitive to oxygen. Upon contact with oxygen, the solution will turn back to pink immediately. This feature allows for easy visual verification of proper anaerobic conditions.

**Figure 4:**
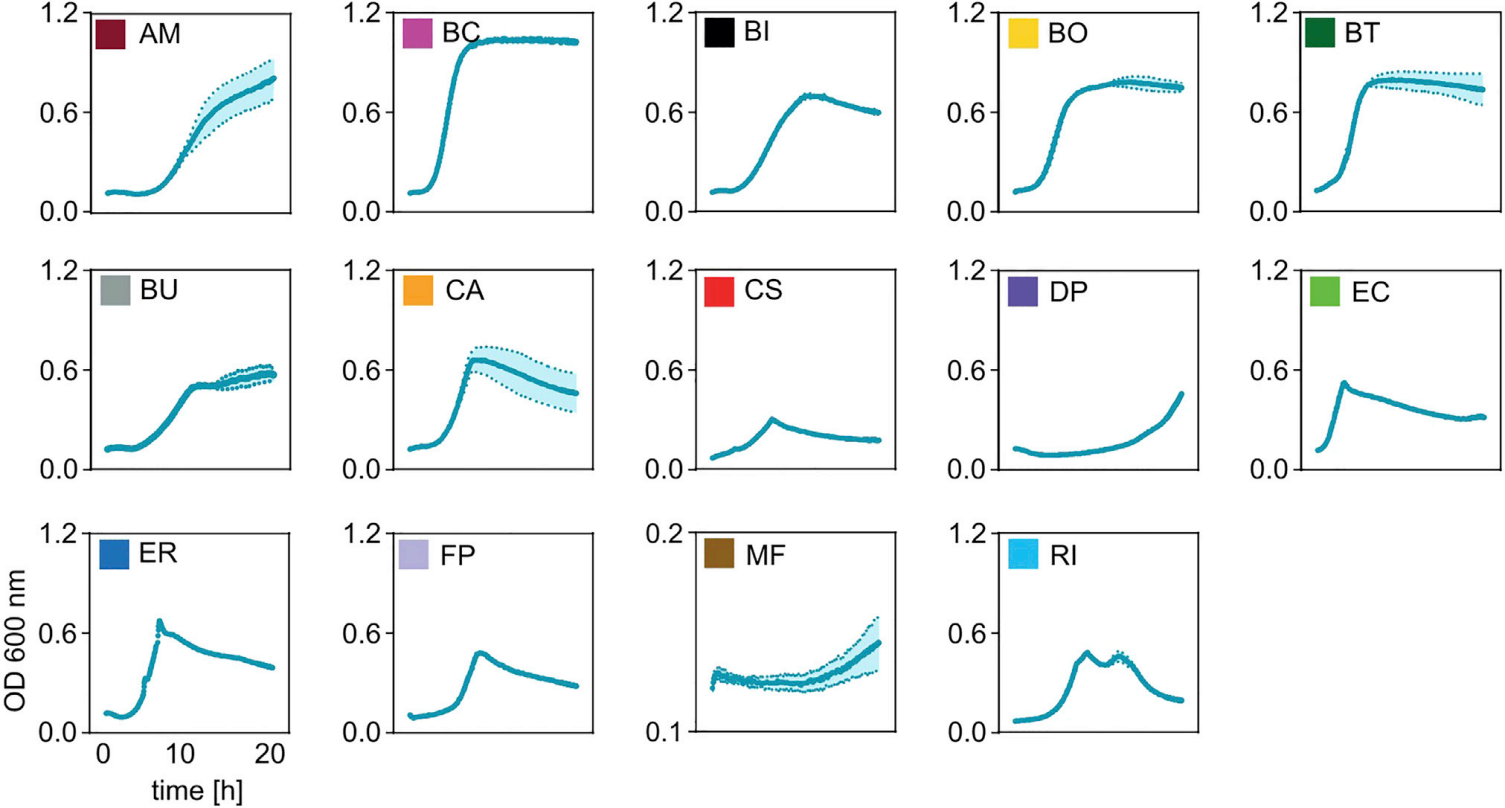
Example growth curves for all 14 strains in mYCFA medium OD at 600 nm depicted as a function of time [h]. Curves were measured by detection of OD at 600 nm (every 2 min) in a 96 well plate and 200 μL mYCFA with equal start ODs of 0.01 at t = 0 for each strain. Blue lines represent mean ODs of 3 independent experiments with shaded blue area indicating SD. Range of x-axis is identical for each panel. For details on bacterial strain abbreviations, see Table 1. Note that ODs of the individual strains could be higher when grown in larger volumes in culture tubes.

Figure 4 depicts example growth curves for all 14 strains in 200 μL mYCFA medium in a 96 well plate, illustrating that all 14 of these human commensals can be properly and efficiently cultured in this medium. To demonstrate proper culturability of all strains, we inoculated 50 μL of the cryostocks of each strain into 2.5 mL mYCFA medium and incubated them for 16 h and 37°C under anaerobic conditions. Next, we detected optical density of these over-night cultures by measuring OD_600_ of 200 μL of each suspension in a transparent 96-well flat bottom plate. After calculation of dilution factors for each culture to achieve an OD_600_ of 0.01, 200 μL of mYCFA was inoculated with the calculated volume of each over-night culture in technical triplicates and incubated for 20 h at 37°C under anaerobic conditions. OD_600_ of non-inoculated mYCFA was used as a BLANK control. Although not all strains grow to the same OD, the resulting growth curves (Figure 4) demonstrate proper culturability of all strains in mYCFA medium.

### Part B: Bacterial culturing in complete mYCFA medium

**Timing: 30 min daily for a period of 3 days**

**Figure 5:**
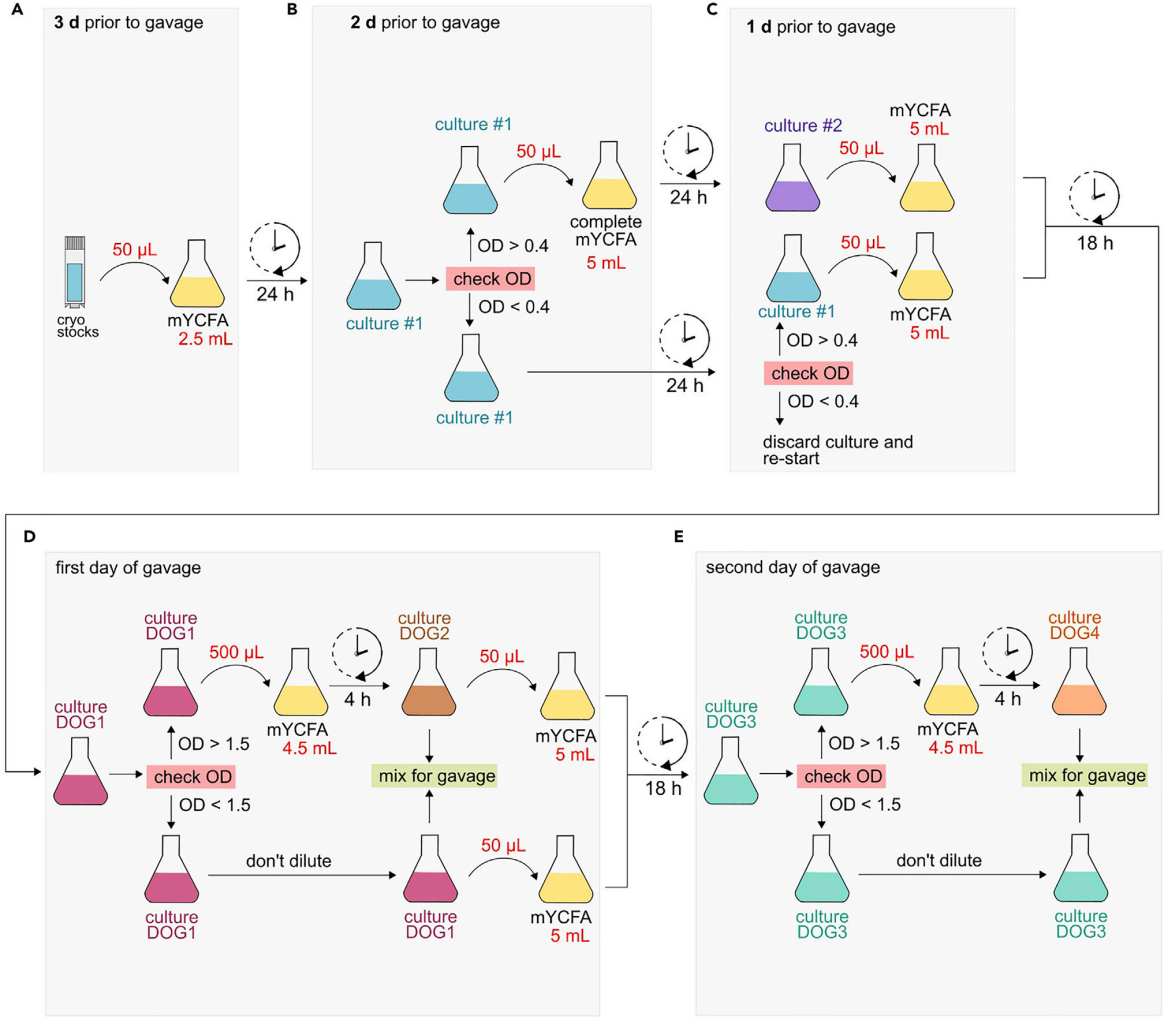
Flowchart for cultivation of 14 human commensals in mYCFA medium Note that the flasks shown in the figures are only for the representative purpose; we used 23 mL culturing tubes with a plastic cap. (A) Inoculation of 50 μL cryo-preserved bacterial stock solution into 2.5 mL mYCFA, followed by 24 h anaerobic incubation at 37°C. Resulting bacterial culture is named “culture #1”. (B) Check OD at 600 nm for every “culture #1” with further actions depending on resulting OD. If OD_600_ is lower than 0.4, culture #1 needs to be incubated for another 24 h (lower panel). If OD_600_ is higher than 0.4, 50 μL of culture #1 is inoculated into 5 mL of fresh mYCFA medium and incubated for 24 h, resulting in “culture #2” (upper panel). Discard culture #1 of these strains. (C) Inoculate 50 μL of “culture #2” into 5 mL of fresh mYCFA medium and incubate for 24 h, resulting in culture “DOG1” (upper panel). Check the OD_600_ of the remaining “culture #1” suspensions. If OD_600_ is higher than 0.4 inoculate 50 μL into 5 mL of fresh mYCFA medium and incubate for 18 h. Also name this culture “DOG1”. If OD_600_ is lower than 0.4 (unlikely), culture should be discarded and not used further. (D) At the day of first gavage, check OD_600_ of all cultures labeled “DOG1”. The target OD of all cultures for the final gavage mix is 0.5 ≤ OD_600_ ≤ 2.0. Thus, all DOG1 cultures providing an OD_600_ of > 1.5 should be diluted and cultured for 4 more hours and be named “DOG2” with their corresponding DOG1 cultures being discarded. All remaining DOG1 cultures will be further incubated for the same 4 h. At the end of this 4 h incubation time, OD_600_ of all DOG2 and remaining DOG1 cultures should be measured to verify that none of these cultures provide an OD_600_ of more than 2.0 or less than 0.5. To prepare cultures for the second gavage mix on the following day, 50 μL of all DOG2 and DOG1 cultures used to prepare gavage mix 1 are to be inoculated into 5 mL and incubated for another 18 h. (E) Repeat steps as explained in (D).

In this step, bacterial suspensions will be cultured from bacterial cryostocks, which are stored at −80°C. Since bacterial growth is strain- and aliquot-dependent, definite growth rates are impossible to precisely predict. Thus, we established a culturing flow chart (Figure 5), which allows for proper cultivation of all 14 strains described in this protocol, independent of individual differences. To ensure sufficient availability of all cultured strains at the day of the intragastric gavage, we recommend starting bacterial cultivation 3 d prior to day 1 of gavage (DOG1).

**CRITICAL:** To avoid any potential cross-contamination, we recommend to properly clean all pipettes and the gloves with a suitable disinfectant before handling the cultures and in between handling different cultures.

3 d prior to intragastric gavage (Figure 5A):

12. Transfer bacterial cryostocks into anaerobic chamber.
13. Verify that complete mYCFA medium displays a yellow color, indicating proper oxygen-reduction.
14. Distribute 2.5 mL of mYCFA into sterile bacterial culturing tubes (one culture tube for each strain). Prepare an additional tube containing only medium without adding any bacteria (BLANK control for OD detection).
15. Label tubes properly (“culture #1”) and inoculate each tube with 50 μL of the respective bacterial cryostock.
16. Vortex tubes briefly and incubate at 37°C for 24 h.

**Figure 6:**
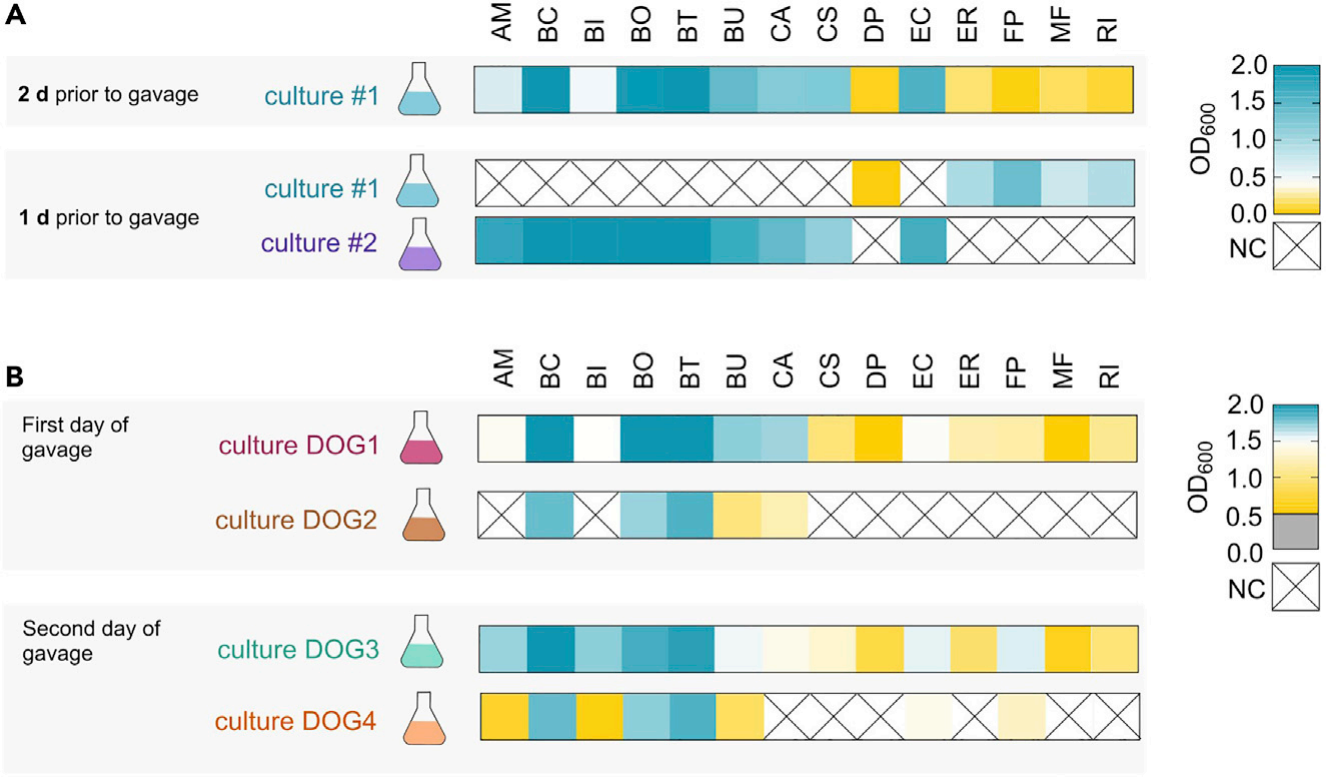
Optical densities of bacterial cultures during culturing procedure according to flowchart in Figure 5 Heatmaps of the optical densities at 600 nm (OD_600_) of “culture #1”, “culture #2”, “DOG1”, “DOG2”, “DOG3” and “DOG4”. Culturing of all 14 depicted strains was performed according to the flowchart in Figure 5 and the same culture nomenclature was used. Underlying primary data are listed in Table 5. (A) OD_600_ of all 14 bacterial cultures 2 days (d) and 1 d before first gavage. OD_600_ ≤ 0.4 are pictured in yellow shades, while OD_600_ > 0.4 are shown in blue shades. (B) OD_600_ of all 14 bacterial cultures on the days of first and second gavage. OD_600_ values of 0.5 < OD_600_ ≤ 1.5 are pictured in yellow shades, while OD_600_ > 1.5 are depicted in blue shades. OD_600_ < 0.5 would be indicated in gray (criterion for not using culture for a gavage mix). In both panels, a black cross on white background indicates that no culture (NC) was generated due to the OD thresholds mentioned in Figure 5.

2 d prior to intragastric gavage (Figure 5B):

17. After 24 h of incubation, check the OD of culture #1 at 600 nm using the non-inoculated tube as a BLANK control. Please note that all ODs mentioned in the example below were detected using a “Biochrom Ultrospec 10 cell density meter”, which is suitable to directly detect ODs in 23 mL glass culture tubes. In case other photometers are used, i.e., photometers that require transferring bacterial suspensions to a detection cuvette, resulting ODs might be slightly different. However, for detection of ODs using a cuvette with a 1 cm light path, all the threshold ODs recommended below remain valid. We do not recommend determining threshold ODs using a 96-well plate and a plate-reader due to heavily different light path lengths and thus restricted comparability to the threshold ODs listed in this protocol.
18. The subsequent steps for each culture depend on the detected OD_600_ of culture #1 (Example: See Figure 6 and Table 6 for example results).
  a. OD_600_ of culture #1 is higher than 0.4:
    Take 50 μL of culture #1 and add to 5 mL of fresh, oxygen-reduced mYCFA medium. Label this culture as “culture #2” and discard the remainder of culture #1. Incubate culture #2 for 24 h.
    (Example: According to Figure 6 and Table 6, this step was applied to cultures of the following strains: AM, BC, BI, BO, BT, BU, CA, CS, and EC)
  b. OD_600_ of culture #1 is lower than 0.4:
    Incubate this culture for another 24 h without addition of medium.
    (Example: According to Figure 6 and Table 6, this step was applied to cultures of the following strains: DP, ER, FP, MF and RI)

**Table 6:** Example OD_600_ of all strains at different stages of cultivation

Strain	Culture #1	Culture #1	Culture #2	Culture DOG1	Culture DOG2	Culture DOG3	Culture DOG4
	2 d prior to gavage	1 d prior to gavage	1 d prior to gavage	1^st^ day of gavage	1^st^ day of gavage	2^nd^ day of gavage	2^nd^ day of gavage
AM	0.64	NC	1.75	1.45	NC	1.71	0.69
BC	2.00	NC	2.00	2.00	1.81	2.00	1.82
BI	0.47	NC	1.99	1.49	NC	1.73	0.59
BO	1.95	NC	2.00	2.00	1.71	1.89	1.73
BT	1.97	NC	2.00	2.00	1.86	1.95	1.86
BU	1.45	NC	1.67	1.73	1.00	1.53	0.90
CA	1.21	NC	1.44	1.70	1.20	1.42	NC
CS	1.22	NC	1.11	0.98	NC	1.32	NC
DP	0.05	0.02	NC	0.54	NC	0.79	NC
EC	1.55	NC	1.63	1.47	NC	1.55	1.43
ER	0.18	0.93	NC	1.19	NC	0.93	NC
FP	0.04	1.38	NC	1.17	NC	1.57	1.28
MF	0.15	0.74	NC	0.54	NC	0.63	NC
RI	0.09	0.89	NC	1.08	NC	0.98	NC

Culture abbreviations refer to abbreviations used in Figure 5.

(NC = not cultured). See Figure 6 for graphical illustration of these values.

1 d prior to intragastric gavage (Figure 5C):

19. After 24 h of incubation, check the OD_600_ of culture #1 for strains treated as described in step 18b. OD_600_ of culture #2 for strains treated as described in step 18a do not need to be checked since proper growth of this strain was already validated the day before. Again, use the non-inoculated tube as a BLANK control.
The subsequent steps are dependent on the culture’s absorbance value:
  a. culture #1 (example: DP, ER, FP, MF and RI)
    Verify that these cultures, which have been incubating for 2 d, finally reach a threshold OD_600_ of 0.4. (See Figure 6A and Table 6 for example results. In this example, culture #1 of ER, FP, MF and RI have reached the threshold OD_600_. DP has not yet reached this threshold.)
    Add 50 μL of culture #1 and to 5 mL fresh complete mYCFA medium (Figure 5C, lower panel). Label the new culture as DOG1 and incubate for 18 h.
    ***Note:*** Culturing of DP to an OD_600_ of > 0.4 can take up to 3 d. In case DP has not yet reached OD_600_ = 0.4 at this stage, continue incubation of DP culture #1 until day of gavage.
    In case any culture #1, other than DP, still yields an OD_600_ < 0.4, these cultures should be discarded and fresh inoculation with a new cryostock should be performed (see also: troubleshooting 1)
  b. culture #2 (example: AM, BC, BI, BO, BT, BU, CA, CS and EC; Figure 6A). Add 50 μL of culture #2 and to 5 mL fresh complete mYCFA medium (Figure 5C, upper panel). Label culture as DOG1 and incubate for 18 h.

First day of gavage (Figure 5D):

20. After 18 h of incubation, check the OD_600_ of culture DOG1 using the non-inoculated mYCFA tube as a BLANK control. Subsequent steps depend on the detected OD_600_ of culture DOG1. See Figure 6B and Table 6 for example values.
  a. OD_600_ of culture DOG1 > 1.5: (example: BC, BO, BT, BU and CA; Figure 6B)
    Add 500 μL of culture DOG1 and to 4.5 mL fresh complete mYCFA medium (Figure 5D, upper panel). Label the culture as DOG2 and incubate for 4 h.
  b. OD_600_ of culture DOG1 < 1.5: (example: AM, BI, CS, DP, EC, ER, FP, MF and RI; Figure 6B). No further dilution. Incubate culture DOG1 for 4 more hours.

21. After 4 h of incubation, check the OD_600_ of DOG1 and DOG2 using the non-inoculated tube as a BLANK control. Cultures should yield an OD_600_ between 0.6 and 1.5.
22. For each mouse to be gavaged, determine the volume of gavage mix per individual (V_G_).

***Note:*** For mice aged 6 weeks or older, we recommend to use 0.2 mL for gavage per mouse (V_G_ = 0.2 mL), based on a minimum animal weight of 20 g. However, the gavage volume for each individual mouse can deviate from the recommended 0.2 mL, depending on the weight of the mice and/or local regulations. Nevertheless, changing the volume of the gavage mix by ± 50% is not expected to negatively affect the colonization efficiency.

23. Calculate the required total volume of gavage mix needed (V_1_) using the following equation, with V_G_ = gavage volume per mouse in mL and n = number of mice to be gavaged:

$$V_{1}\;\left[mL\right]=\left(V_{G}\;x\;n\right)+2$$

(Example: for 15 mice to be gavaged, prepare:

$$V_{1}=\left(0.2\;x\;15\right)+2=5.0\;mL$$

of the gavage mix)

24. Prepare a mix containing equal volumes of all bacterial cultures.
  ***Note:*** Adjusting the required volumes for each culture according to the detected OD_600_ is not necessary. We could previously confirm that varying bacterial numbers in different gavage mixes result in comparable relative abundances of all gavaged strains in mice with the same genetic background when following the protocol described above.
  a. Calculate the required volume of each culture (V_2_) using the following formula with z = number of strains to be gavaged and V_1_ which was determined in step 23.
    $$V_{2}\left[mL\right]=\;\frac{V_{1}}{z}$$
    (Example: for 14 strains to be gavaged into 15 mice, prepare:
    $$V_{2}\left[mL\right]=\;\frac{5.0\;mL}{14}=0.357\;mL=357\;\mu L$$
    of each culture)
  b. Add the volume to be used from each DOG2 culture (V_2_) into a single sterile 15 mL (or 50 mL) tube. Use the respective DOG1 culture when no culture DOG2 was prepared (according to criteria mentioned in step 20). See Table 7 for examples.

**Table 7:** Example pipetting scheme for creating a gavage mix

Strain	Culture	Volume
AM	DOG1	357 μL
BC	DOG2	357 μL
BI	DOG1	357 μL
BO	DOG2	357 μL
BT	DOG2	357 μL
BU	DOG2	357 μL
CA	DOG2	357 μL
CS	DOG1	357 μL
DP	DOG1	357 μL
EC	DOG1	357 μL
ER	DOG1	357 μL
FP	DOG1	357 μL
MF	DOG1	357 μL
RI	DOG1	357 μL

25. Transfer the gavage mix into a gnotobiotic facility.
  ***Note:*** It is not necessary to perform the transport of the gavage mix tube under anaerobic conditions. However, the gavage mix should be gavaged as soon as possible after its preparation and removal from the anaerobic chamber.

26. Perform the first intragastric gavage as described below:
  ***Note:*** Freeze the remaining gavage mix at −20°C so that the composition of the gavage mix can be checked or verified.
  a. Transfer the gavage mix, a gavage needle (gauge 20, straight 38 mm) and sterile 1 mL plastic syringes into the gnotobiotic animal facility.
  b. Determine weight of each mouse prior to performing the gavage in order to monitor possible weight loss due to complications during the gavage.
  c. Attach the gavage needle onto the 1 mL syringe and fill the syringe with 0.6 mL of bacterial suspension (sufficient to gavage three animals).
    ***Note:*** The bacterial gavage mix will change color when exposed to oxygen and might therefore turn purple when tube is opened under the laminar flow.
  d. Restrain the mouse and gavage 0.2 mL of the bacterial gavage mix intragastrically.

27. Add 500 μL of each bacterial culture (DOG1 or DOG2), which was used for preparation of the gavage mix for the first intragastric gavage, to 4.5 mL of fresh mYCFA medium. Label the culture as DOG3 and incubate for 18 h.

Second day of gavage (Figure 5E)

28. After 18 h of incubation, check OD_600_ of culture DOG3 using the non-inoculated mYCFA tube as a BLANK control. Subsequent steps depend on the detected OD_600_ of culture DOG3. See Figure 6B and Table 6 for example values.
  a. OD_600_ of culture DOG3 > 1.5: (example: AM, BC, BI, BO, BT, BU, EC, and FP; Figure 6B)
    Add 500 μL of culture DOG3 and to 4.5 mL fresh complete mYCFA medium (Figure 5D, upper panel). Label the culture as DOG4 and incubate for 4 h.
  b. OD_600_ of culture DOG3 < 1.5: (example: CA, CS, DP, ER, MF and RI; Figure 6B). No further dilution. Incubate culture DOG3 for 4 more hours.

29. After 4 h of incubation, check the OD_600_ of DOG1 and DOG2 using the non-inoculated tube as a BLANK control. Cultures should yield an OD_600_ between 0.6 and 1.5. Repeat steps 22 to 26 as described above.

### Part C: Evaluation of colonization success

Although establishing a relatively stable microbiota composition will take up to 20 d, it is possible to evaluate the success of bacterial colonization as early as 5 days after initial gavage by collecting fecal samples from gavaged mice and isolating the bacterial DNA as described below.

30. Prepare one sterile, DNase-free 2 mL screw cap tube for each gavaged mouse.
31. Collect at least 1 fecal pellet of roughly 20 mg per mouse.
32. Freeze the collected fecal samples at −20°C or directly proceed with step 33.

**Pause point:** The following steps describe the extraction of bacterial DNA from mouse fecal samples using phenol:chloroform:isoamyl alcohol (25:24:1), followed by purification of DNA with the QIAGEN DNeasy Blood & Tissue kit. We recommend isolating bacterial DNA from fecal samples as described below. However, use of other commercially available kits could also be used in place of steps 37 to 55, provided that the replacement protocol can reliably isolate bacterial DNA from mouse fecal samples.

Before starting the extraction procedure, prepare 3 × 1.5 mL sterile Eppendorf tubes for each fecal sample.

**CRITICAL:** All steps using phenol:chloroform:isoamyl alcohol (25:24:1) or chloroform must be performed under a laboratory fume hood.

33. If not done previously, transfer each fecal sample to a 2 mL screwcap tube and add:
  a. The equivalent of roughly 250 μL of acid-washed glass beads (212–300 μm)
  b. 500 μL buffer A (DNA extraction component 1)
  c. 210 μL 20% SDS (DNA extraction component 2)
  d. 500 μL phenol:chloroform:isoamyl alcohol (25:24:1), pH 8.0

***Note:*** Pre-combine Buffer A and 20% SDS in the appropriate ratio, mix well and then add 710 μL of the buffer A/SDS mixture to each tube.

34. Bead-beat the mixture on the highest frequency (30 Hz) for 3 min using a bead mill (make sure to balance rotor with a minimum of 4 tubes).
35. Centrifuge at 18000 × *g* and 4°C for 3 min.
36. Transfer the aqueous phase (phase on the top) into a new 1.5 mL sterile Eppendorf tube.
37. Add 500 μL phenol:chloroform:isoamyl alcohol (25:24:1) and mix tube by inversion.
38. Centrifuge at 18000 × *g* and 4°C for 3 min.
39. Transfer the aqueous phase (phase on the top) into a new 1.5 mL sterile Eppendorf tube.
40. Add 500 μL of 100% chloroform and mix by inversion.
41. Centrifuge at 18000 × *g* and 4°C for 3 min.
42. Transfer aqueous phase (top one) into a new 1.5 mL sterile Eppendorf tube
43. Add 60 μL 3 M sodium acetate pH 5.5 (DNA extraction component 3)
44. Add 600 μL 100% isopropanol
45. Mix by inversion and incubate at −20°C for at least 1 h to precipitate DNA.

***Note:*** To save time, incubate samples at −80°C for at least 20 minutes instead.

**Pause point:** It is possible to store samples at −20°C for up to 24 h.

46. Centrifuge at maximum speed (21000 × *g*) and 4°C for 20 min.
47. Discard supernatant into the sink or a liquid waste container by inverting the tube.

***Note:*** In case the pellet detaches from the tube bottom, use a pipette to carefully remove supernatant.

48. Add 1 mL of 70% ethanol.
49. Centrifuge at 18000 × *g* and 4°C for 3 min.
50. Discard supernatant as described in step 47.
51. Dry the pellet for 1 h at RT.
52. Resuspend dried pellet in 100 μL of nuclease-free water (Carl Roth).
53. Store at −20°C until further use or continue with step 54.

54. Extracted DNA samples will now be purified to remove remaining RNA and proteins for further downstream applications. For this purpose, we recommend using the QIAGEN DNeasy Blood & Tissue kit with some modifications as outlined below:
  a. Add 300 μL QIAGEN Buffer ATL and 4 μL RNase A to the 100 μL DNA solution (see step 52).
  b. Incubate at RT for 2 min.
  c. Add 400 μL Buffer AL.
  d. Add 40 μL QIAGEN proteinase K.
  e. Incubate at 56°C for 30 min.
  f. Add 40 μL 3 M sodium acetate pH 5.5.
  g. Add 440 μL 100% ethanol.
  h. Mix well by pipetting and transfer 750 μL on a QIAGEN DNeasy column.
  i. Centrifuge at 8000 × *g* for 1 min and discard flow-through.
  j. Transfer the remaining volume onto the same QIAGEN DNeasy column.
  k. Centrifuge at 8000 × *g* for 1 min and discard the flow-through.
  l. Add 500 μL buffer AW1 and centrifuge 8000 × *g* for 1 min and discard flow-through.
  m. Add 500 μL buffer AW2, centrifuge at maximum speed for 3 min and discard the flow-through.
    ***Optional:*** Repeat centrifugation without addition of any buffer at max speed to remove remaining ethanol from the samples.
  n. Discard the collection tube and place the column on a new 1.5 mL tube.
  o. Add 50 μL of DNase & RNase free water onto the column membrane.
  p. Centrifuge at 8000 × *g* for 1 min.

55. Resulting purified fecal DNA solutions can be stored at −20°C for at least 6 months.

56. For subsequent determination of relative abundances of bacterial strains present in the fecal samples, generation of standards of purified DNA from each strain is required.
  a. Inoculate 50 μL of each strain into separate tubes containing 2.5 mL of mYCFA medium and incubate for 24 h at 37°C under anaerobic conditions (see steps 12 to 16).
  b. After 24 h of incubation, check OD of all cultures at 600 nm using the non-inoculated tube as a BLANK control.
  c. The subsequent steps to be carried out for each culture depend on the detected OD_600_
    i. OD_600_ > 0.4:
      Add 50 μL of culture #1 and to 5 mL of fresh, oxygen-reduced mYCFA medium. Incubate for 24 h.
    ii. OD_600_ < 0.4:
      Incubate the culture for another 24 h without further dilution
  d. After 24 h of incubation, verify that OD_600_ is > 1.0 for all cultures. If not, continue incubation for another 24 h without dilution.
    ***Note:*** From this step on, working under anaerobic conditions is not necessary.
  e. Centrifuge bacterial cultures for 10 min at 10000 × *g*.
  f. Discard supernatant and resuspend pellet in 500 μL of buffer A.
  g. Isolate DNA by following the instructions described in steps 33 to 55.
  h. Detect DNA concentration, e.g., using Nanodrop, and create 4 standards with specific DNA concentrations as listed in Table 8.

**Table 8:** Concentrations of DNA standards for qPCR based detection of relative bacterial abundances

Standard	Concentration
Standard 1	20 ng μL^−1^
Standard 2	2 ng μL^−1^
Standard 3	0.4 ng μL^−1^
Standard 4	0.01 ng μL^−1^
  i. Standards can be stored at −20°C for at least 6 months.

57. The DNA extraction is followed by qPCR for detection and quantification of 14 bacterial strains using strain-specific primer pairs. For details on strain-specific primer sequences, see Table 9.
  ***Note:*** Primers are specific to regions that only occur once in the respective bacterial genome, resulting in an amplicon-to-genome ratio of 1:1. Target genes for each primer pair are listed in Table 10.

**Table 9:** List of bacterial strain-specific primer sequences and corresponding melting temperature of resulting amplicons

Strain/primer pair	Forward primer		Reverse primer		T_M_
	Primer name	Sequence (5’-->3′)	Primer name	Sequence (5’-->3′)	
AM	AM F	GACCGGCATGTTCAAGCAGACT	AM R	AAGCCGCATTGGGATTATTTGTT	85.5°C
BC	BC F	GGCGCATGACATTGGAGGTTT	BC R	AATACGCCGCATCGCTTTTTC	81.6°C
BI	BI F	ACCGGATTCCTATATTGGGCAGTC	BI R	TTCGCTTTTGGCTCTTCCTATTTTC	84.3°C
BO	BO F	GTGAAGGTGCCATCGGAGGAC	BO R	GGACGCTTTGGCCACTATTTCA	83.4°C
BT	BT F	TACTCGCCTCTTTGCAACCCTACC	BT R	GGCCCCAGATCCGAACAACAC	82.8°C
BU	BU F	GCTACCGGGAGATACTGGATTGG	BU R	TGCGGCGGCCTTTGAAC	84.3°C
CA	CA F	GTTCGCGTTCGTTATGGTTGGT	CA R	GTTGAGCTGGGCCGATTGTG	89.4°C
CS	CS F	CCGCTTGGCATGAAACAGGTATC	CS R	TTGGAAGCGGCGAAGAATGG	80.1°C
DP	DP F	TGGCTTCAGGCAAATCTCAAAT	DP R	TCCGGGGAATCAAAACCATAC	83.1°C
EC	EC F	GGTGGCTGGGTGATGTAAAACTGA	EC R	ACCGCCGAGCAAAATGAAGC	87.0°C
ER	ER F	AGCTTGTGCCGCCCATCTCTAT	ER R	TTGCGGTAAAGCTTTGGTGTGG	83.1°C
FP	FP F	TGCCCCCGGGTGGTTCT	FP R	CGTTATTCAAAGCCCCGTTATCAA	80.1°C
MF	MF F	CAGGGATTTTACGTGCTTTATTTTAGTTAT	MF R	AGTTCGGATTCGCTCGTATTTTCT	78.6°C
RI	RI F	TCGAAATTAAAGAGACGGAAACAGAAG	RI R	CCGCTCATATCAATCGAAAACACA	81.9°C

**Figure 7:**
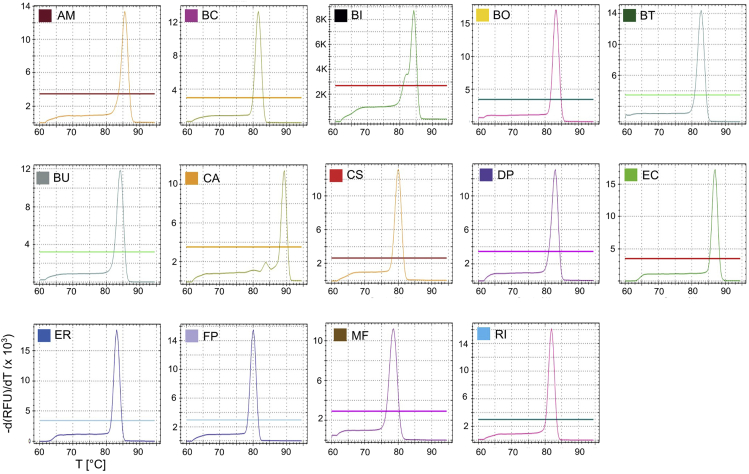
qPCR melting curves using strain-specific primer pairs Fecal samples of gnotobiotic mice colonized with 14 different human commensals were subjected to DNA isolation followed by qPCR using strain-specific primer pairs (see also Table 9). Representative melting curves of amplicons corresponding to each primer pair are shown. Table 9 also lists an overview of reference melting temperatures for each amplicon.
  c. Run the qPCR with the specifications as listed in Table 12.

58. The following protocol describes how to perform the qPCR using SYBR Green. However, other detection systems can be used instead, according to the researcher’s preference.
  a. Detect DNA concentrations in the fecal DNA solutions obtained from step 55. Dilute samples to a concentration of 20 ng μL^−1^ with ddH_2_O.
  b. Using Platinum *Taq* DNA polymerase and SYBR Green, prepare the reaction mix for each standard dilution and each sample according to the pipetting scheme in Table 11. See Table 12 for qPCR specifications and Table 13 for an example of complete detection of all standards and 1 sample (Fecal 1) and the corresponding Cq values (see quantification and statistical analysis). See Figure 7 for example melting curves of resulting amplicons.

**Table 11:** Example pipetting scheme for qPCR detection

Reagent/component	Concentration per reaction	Volume per sample
DNase & RNase H_2_O		8.9 μL
10× PCR Buffer	1×	1.25 μL
MgCl_2_	1.5 mM	0.375 μL
dNTPs	0.2 mM	0.25 μL
Primer Forward	0.2 μM	0.25 μL
Primer Reverse	0.2 μM	0.25 μL
SYBR™ Green I Nucleic Acid Gel Stain	1×	0.125 μL
Platinum™ Taq DNA Polymerase	0.5 U	0.1 μL
DNA 1	max. 20 ng μL^−1^	1.0 μL
**Total**		**12.5 μL**

1 For fecal DNA solutions: use 20 ng μL^−1^. For bacterial DNA standards, use the concentrations mentioned in Table 7.

**Table 12:** qPCR reaction specifications

Steps	Temperature	Time	Cycles
Pre-denaturation	95°C	3 min	1
Denaturation	95°C	3 s	40
Annealing	55°C	20 s	
Extension	68°C	20 s	
Post-Extension	95°C	15 s	1
Pre-Melting-Curve Cooling Step	60°C	15 s	
Melting Curve	60°C–95°C	Incremental increase of temperature by 0.3°C every 15 s until temperature reaches 95°C	
Storage	20°C	→∞	1

**Table 13:** Example Cq values of a qPCR run including standards for all primer pairs and DNA isolated from 1 sample (“Fecal 1”)

Sample no	Sample type	Sample	Concentration	Forward primer	Reverse primer	Cq
1	Standard	AM culture	20 ng μL^−1^	AM F	AM R	13.71
2	Standard	AM culture	2 ng μL^−1^	AM F	AM R	17.54
3	Standard	AM culture	0.4 ng μL^−1^	AM F	AM R	20.59
4	Standard	AM culture	0.01 ng μL^−1^	AM F	AM R	27.79
5	Standard	BC culture	20 ng μL^−1^	BC F	BC R	14.32
6	Standard	BC culture	2 ng μL^−1^	BC F	BC R	17.96
7	Standard	BC culture	0.4 ng μL^−1^	BC F	BC R	20.99
8	Standard	BC culture	0.01 ng μL^−1^	BC F	BC R	26.97
9	Standard	BI culture	20 ng μL^−1^	BI F	BI R	13.29
10	Standard	BI culture	2 ng μL^−1^	BI F	BI R	16.64
11	Standard	BI culture	0.4 ng μL^−1^	BI F	BI R	20.41
12	Standard	BI culture	0.01 ng μL^−1^	BI F	BI R	27.26
13	Standard	BO culture	20 ng μL^−1^	BO F	BO R	14.65
14	Standard	BO culture	2 ng μL^−1^	BO F	BO R	18.28
15	Standard	BO culture	0.4 ng μL^−1^	BO F	BO R	21.94
16	Standard	BO culture	0.01 ng μL^−1^	BO F	BO R	28.63
17	Standard	BT culture	20 ng μL^−1^	BT F	BT R	15.48
18	Standard	BT culture	2 ng μL^−1^	BT F	BT R	19.21
19	Standard	BT culture	0.4 ng μL^−1^	BT F	BT R	22.33
20	Standard	BT culture	0.01 ng μL^−1^	BT F	BT R	28.53
21	Standard	BU culture	20 ng μL^−1^	BU F	BU R	15.02
22	Standard	BU culture	2 ng μL^−1^	BU F	BU R	18.30
23	Standard	BU culture	0.4 ng μL^−1^	BU F	BU R	21.68
24	Standard	BU culture	0.01 ng μL^−1^	BU F	BU R	28.29
25	Standard	CA culture	20 ng μL^−1^	CA F	CA R	16.12
26	Standard	CA culture	2 ng μL^−1^	CA F	CA R	19.27
27	Standard	CA culture	0.4 ng μL^−1^	CA F	CA R	22.27
28	Standard	CA culture	0.01 ng μL^−1^	CA F	CA R	29.78
29	Standard	CS culture	20 ng μL^−1^	CS F	CS R	17.22
30	Standard	CS culture	2 ng μL^−1^	CS F	CS R	20.54
31	Standard	CS culture	0.4 ng μL^−1^	CS F	CS R	24.62
32	Standard	CS culture	0.01 ng μL^−1^	CS F	CS R	30.36
33	Standard	DP culture	20 ng μL^−1^	DP F	DP R	14.18
34	Standard	DP culture	2 ng μL^−1^	DP F	DP R	17.33
35	Standard	DP culture	0.4 ng μL^−1^	DP F	DP R	20.63
36	Standard	DP culture	0.01 ng μL^−1^	DP F	DP R	28.50
37	Standard	EC culture	20 ng μL^−1^	EC F	EC R	14.11
38	Standard	EC culture	2 ng μL^−1^	EC F	EC R	18.24
39	Standard	EC culture	0.4 ng μL^−1^	EC F	EC R	21.00
40	Standard	EC culture	0.01 ng μL^−1^	EC F	EC R	27.23
41	Standard	ER culture	20 ng μL^−1^	ER F	ER R	14.24
42	Standard	ER culture	2 ng μL^−1^	ER F	ER R	17.99
43	Standard	ER culture	0.4 ng μL^−1^	ER F	ER R	20.79
44	Standard	ER culture	0.01 ng μL^−1^	ER F	ER R	26.87
45	Standard	FP culture	20 ng μL^−1^	FP F	FP R	13.03
46	Standard	FP culture	2 ng μL^−1^	FP F	FP R	16.65
47	Standard	FP culture	0.4 ng μL^−1^	FP F	FP R	21.26
48	Standard	FP culture	0.01 ng μL^−1^	FP F	FP R	26.58
49	Standard	MF culture	20 ng μL^−1^	MF F	MF R	16.25
50	Standard	MF culture	2 ng μL^−1^	MF F	MF R	19.96
51	Standard	MF culture	0.4 ng μL^−1^	MF F	MF R	22.32
52	Standard	MF culture	0.01 ng μL^−1^	MF F	MF R	29.22
53	Standard	RI culture	20 ng μL^−1^	RI F	RI R	14.43
54	Standard	RI culture	2 ng μL^−1^	RI F	RI R	18.51
55	Standard	RI culture	0.4 ng μL^−1^	RI F	RI R	21.58
56	Standard	RI culture	0.01 ng μL^−1^	RI F	RI R	28.97
57	Sample	Fecal 1	NA	AM F	AM R	17.93
58	Sample	Fecal 1	NA	BC F	BC R	19.62
59	Sample	Fecal 1	NA	BI F	BI R	20.73
60	Sample	Fecal 1	NA	BO F	BO R	16.81
61	Sample	Fecal 1	NA	BT F	BT R	18.15
62	Sample	Fecal 1	NA	BU F	BU R	17.56
63	Sample	Fecal 1	NA	CA F	CA R	23.52
64	Sample	Fecal 1	NA	CS F	CS R	20.26
65	Sample	Fecal 1	NA	DP F	DP R	24.39
66	Sample	Fecal 1	NA	EC F	EC R	20.40
67	Sample	Fecal 1	NA	ER F	ER R	20.05
68	Sample	Fecal 1	NA	FP F	FP R	31.88
69	Sample	Fecal 1	NA	MF F	MF R	20.24
70	Sample	Fecal 1	NA	RI F	RI R	22.31

**Table 10:** List of target gene sequences for strain-specific primer design

Strain/primer pair	Target strain collection number	Taxonomy ID	Reference sequence	Target gene locus tag
AM	*Akkermansia muciniphila* ATCC BAA-835	NCBI:txid349741	NC_010655	Amuc_1599
BC	*Bacteroides caccae* ATCC 43185	NCBI:txid411901	NZ_AAVM02000002	BACCAC_01370
BI	*Barnesiella intestinihominis* YIT 11860	NCBI:txid742726	ADLE01000001	HMPREF9448_00112
BO	*Bacteroides ovatus* ATCC 8483	NCBI:txid411476	NZ_AAXF02000051	BACOVA_03510
BT	*Bacteroides thetaiotaomicron* VPI-5482	NCBI:txid226186	NC_004663	BT4272
BU	*Bacteroides uniformis* ATCC 8492	NCBI:txid411479	NZ_AAYH02000049	BACUNI_04564
CA	*Collinsella aerofaciens* ATCC 25986	NCBI:txid411903	NZ_AAVN02000001	COLAER_00143
CS	*Clostridium symbiosum* ATCC 14940	NCBI:txid411472	CLOSYM-1.0_Cont1178.4	CLOSYM_03515
DP	*Desulfovibrio piger* ATCC 29098	NCBI:txid411464	NZ_ABXU01000029	DESPIG_01169
EC	*Escherichia coli* HS	NCBI:txid331112	NC_009800	EcHS_A1069
ER	*Eubacterium rectale* DSM 17629	NCBI:txid657318	FP929042	EUR_02900
FP	*Faecalibacterium prausnitzii* A2-165	NCBI:txid411483	NZ_ACOP02000003	FAEPRAA2165_00192
MF	*Marvinbryantia formatexigens* DSM 14469	NCBI:txid478749	NZ_ACCL01000097	BRYFOR_02731
RI	*Roseburia intestinalis* L1-82	NCBI:txid536231	NZ_ABYJ01000040	ROSINTL182_00420

## Expected outcomes

Reference melting curves of the amplicons using primer pairs listed in Table 9 are depicted in Figure 7.

**Figure 8:**
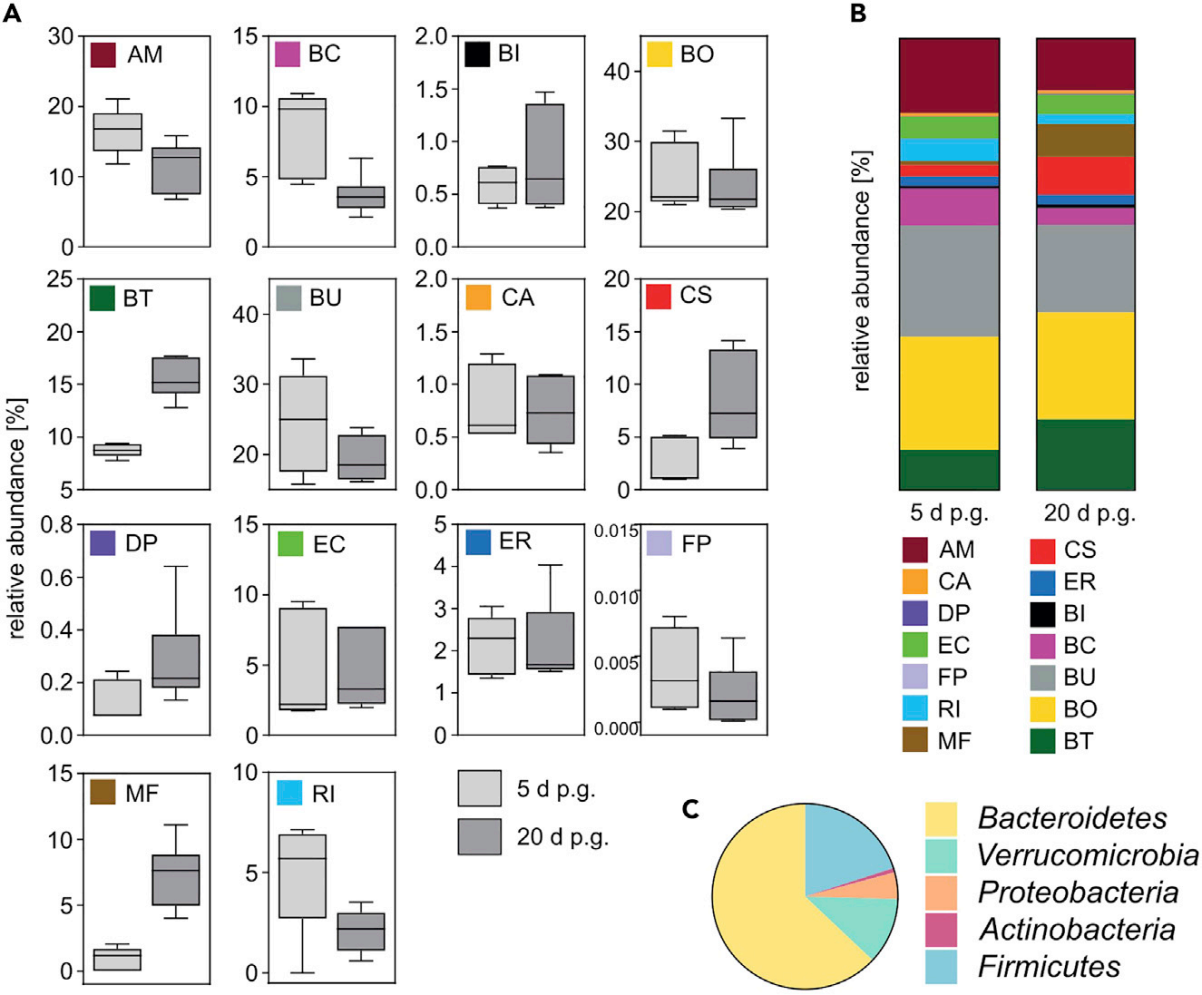
Relative abundances of bacterial strains in fecal samples on different timepoints after intragastric gavage Fecal samples from mice that were gavaged with a 14 strain mix were collected either 5 d (5 d p.g.) or 20 d (20 d p.g.) after first intragastric gavage. Samples were subjected to DNA isolation, as described in this protocol, and qPCR using strain-specific primer pairs was performed to determine relative bacterial abundances. (A) Relative bacterial abundances in percent of total bacteria for each individual strain detected from fecal samples collected on either 5 d (5 d p.g.) (n = 5) or 20 d (20 d p.g.), (n = 5) after first intragastric gavage. Box plots depict mean, highest and lowest value, as well as the 90% confidence interval. (B) Mean relative abundances of all 14 bacterial strains in percent of total bacteria from fecal samples collected on either 5 d p.g or 20 d p.g. Data shown in (B) originate from the same data sets as shown in (A). (C) Phylum-level microbiome composition 20 d after initial gavage. Data shown in (C) originate from the same data sets as (A). See Table 1 for more information on phylum classification of each strain.

In this protocol, we recommend collecting fecal samples for colonization verification 5 days after initial gavage. This is meant to accelerate the experimental timeline, since successful colonization of each strain should be properly verified before starting any procedures or further interventions on the gavaged mice. Although the relative abundance of each of the gavaged strains will slightly change after the first fecal collection, it is nevertheless possible to confirm that each strain has successfully colonized the host. Figure 8 illustrates the differences between the relative bacterial abundances at day 5 and day 20 after initial gavage. Note, for example, that MF was detected only at very low abundance in fecal samples collected 5 d after initial gavage, yet establishes at a typical abundance 20 d after initial gavage. Thus, failing to detect MF in fecal samples collected after 5 d should not be considered a sign of failed colonization as long as it was successfully detected in the gavage mix.

***Note:*** The data shown in Figure 8 were obtained in C57BL/6 mice. We have observed slight differences in the relative abundance of individual strains when mouse chows from distinct suppliers were used or different mouse strains such as Swiss Webster were used (data not shown). Thus, we recommend using a consistent mouse strain and mouse chow for a related set of experiments within a study.

**Figure 9:**
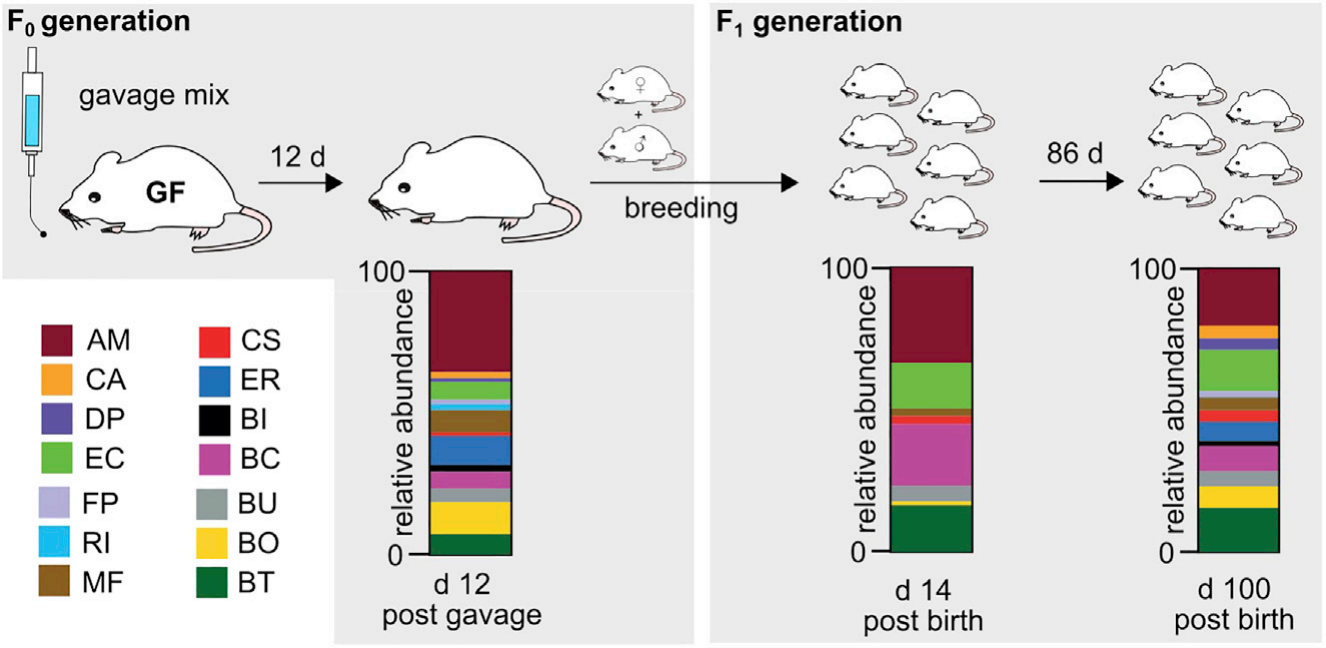
Relative abundances of bacterial strains after maternal transmission during and after weaning period Fecal samples from mice that were gavaged with a 14 strain mix were collected at 12 d (12 d p.g.) after first intragastric gavage and during breeding stages. Samples were subjected to DNA isolation, as described in this protocol, and qPCR using strain-specific primer pairs was performed to determine relative bacterial abundances. Fecal samples from pups were collected either 14 d (14 d p.b.) post birth or 100 d (100 d p.b.) post birth weaned onto two different diets. Stack plots show mean relative abundance of all 14 bacterial strains as a percent of total bacteria from collected fecal samples.

Importantly, this microbiome composition is fully transmissible to offspring of gavaged parent mice (see Figure 9).

## Quantification and statistical analysis

Table 13 lists detected Cq values of an example qPCR run of all pure culture-derived standards and isolated DNA from one example fecal pellet collected from a mouse 20 days after gavage with all 14 strains.

To calculate relative abundances of each strain from these detected Cq values, follow the instructions described below:

1. Perform a linear regression of each standard by plotting the detected Cq value against the logarithm of the DNA concentration and note the linear function of the regression.

**Figure 10:**
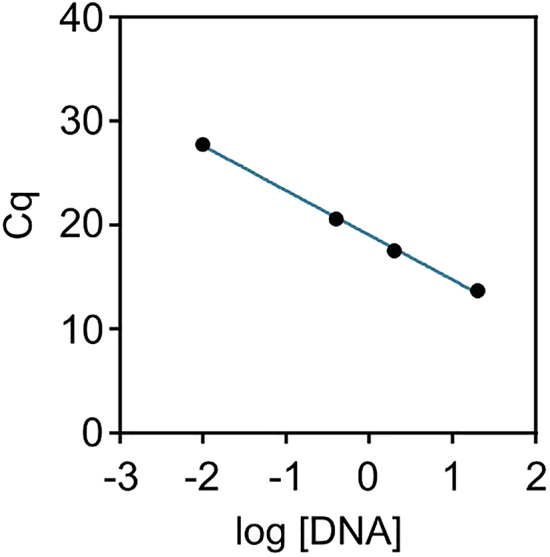
Standard curve of AM-specific DNA amplification Resulting Cq values are plotted against the respective logarithm of the DNA concentration. Underlying values are listed in Table 13.

Example: See Figure 10 for a standard curve for AM-specific amplicons using detected Cq values for AM-specific standards, which are listed in Table 13.

Linear function for this example:

y=−4.29×x+19.05

2. Calculate the starting concentration of bacterial DNA from each strain present in the fecal sample by using the linear regressions determined in the previous step.

Example: Detection of starting concentration of AM bacterial DNA in sample “Fecal 1” using the detected Cq values listed in Table 13.

Cq value using AM-specific primers (sample 57 in Table 13): 17.93.

$$\log\left[DNA\right]=x=\frac{y-19.05}{-4.29}=\frac{17.93-19.05}{-4.29}=\frac{-1.12}{-4.29}=0.261$$

This corresponds to a starting concentration of AM-specific DNA in sample “Fecal 1” of

$$\left[DNA\right]=10^{0.261}=1.82\;\frac{ng}{\mu L}$$

3. Repeat steps 1 and 2 for each primer pair combination.
4. After completion of step 3, sum the bacterial DNA starting quantities (in ng) of each strain to determine the total bacterial DNA starting quantity.

As 1 μL of isolated DNA was used for qPCR, the bacterial DNA starting quantity (Q_S_) is calculated as follows:

$$Q_{S}=\left[DNA\right]\times 1\;\mu L$$

Example: Detection of starting quantity of AM bacterial DNA in sample “Fecal 1” using the calculated starting concentration as determined in step 2.

$$Q_{S}=1.82\;\frac{ng}{\mu L}\times 1\;\mu L=1.82\;ng$$

See Table 14 for a list of each determined bacterial DNA starting quantity and the resulting total bacterial DNA starting quantity using example Cq values listed in Table 13.

5. Calculate relative abundance (rel ab) using the determined strain-specific (Q_S_) and the total (Q_T_) bacterial DNA starting quantity
  $$rel\;ab=\frac{Q_{S}}{Q_{T}}\times 100$$

**Table 14:** List of determined strain-specific and total bacterial DNA starting quantities

Primer pair	Bacterial DNA starting quantity [ng]
AM	1.82
BC	0.8208
BI	0.3112
BO	5.595
BT	4.126
BU	4.007
CA	0.2545
CS	3.33
DP	0.06959
EC	0.5441
ER	0.6022
FP	0.00066
MF	1.71
RI	0.2987
**Total bacterial DNA starting quantity**	**23.489**

Example: The resulting relative abundance of AM in sample “Fecal 1” is:

$$rel\;ab\left(AM\right)=\frac{1.82\;ng}{23.489\;ng}\times 100=7.79\%$$

## Limitations

Certain primers pairs used in this protocol can provide cross-reaction with similar sequences in the genomes of other strains. Although this is not a pronounced problem and rarely occurs, when all strains have properly colonized, melting curves of each primer pair-sample combination should be properly checked.

## Troubleshooting

### Problem 1

OD_600_ of cultures DOG1(DOG3) or DOG2(DOG4) do not reach the required thresholds as described in Figure 5 to create the bacterial gavage mixes (steps 20 and 28).

### Potential solution

Solution 1: OD_600_ < 0.6: Let the cultures grow for 2 more hours and check OD_600_ again.

Solution 2: OD_600_ > 1.5: Add 500 μL of this culture to 4.5 mL of fresh complete mYCFA medium and incubate for 2 h.

### Problem 2

OD_600_ for some or all strains during the start of the cultivation period (d 0 to d 2, Figure 5) is consistently below 0.4 (steps 18 and 19).

### Potential solution

Solution 1: Control the atmosphere within the anaerobic chamber and verify proper oxygen-free conditions.

Solution 2: If some cryostocks do not display sufficient growth, inoculate a new bacterial cryostock into fresh mYCFA medium in order to restart the culture. Use replicates of each bacterial strain in order to have a backup bacterial culture.

Solution 3: Use “use and throw” cryostocks (50 μL volumes) which will avoid freeze-thaw cycles.

## Resource availability

### Lead contact

Further information and requests for resources and reagents should be directed to and will be fulfilled by the lead contact. Mahesh S. Desai; mahesh.desai@lih.lu

### Materials availability

This study did not generate new unique reagents.

### Data and code availability

The published article includes all datasets generated or analyzed during this study.
